# Abstract of the Proceedings of the Buffalo Medical Association

**Published:** 1856-07

**Authors:** Sanford B. Hunt


### BUFFALO MEDICAL JOURNAL

AND

### MONTHLY REVIEW.

**VOL. 12.** JULY, 1856. **NO. 2.**

### ORIGINAL COMMUNICATIONS.

ART. I.—*Abstract of the Proceedings of the Buffalo Medical Association.*

The association met at its room on Tuesday evening, June 3d.

Present—Dr. Eastman (in the chair); Drs. Flint, Lewis, Treat, Strong, Nichols, Hadley, Wyckoff, Holmes, White, Mixer, Howell, Hawley, Rochester, Lemon, Hunt, Gay, Wilcox, Newman and Lay.

Drs. Holmes and Whitehead were proposed for membership.

*Prof. Flint*, as chairman of the committee on Pneumonia, read the following report on the diagnostic value of the buffy coat of the blood:

*Report on the "Diagnostic Value of the Buffy Coat."* By AUSTIN FLINT, M. D., Chairman of Committee. Read at a meeting of the Buffalo Medical Association, June 3d, 1856.

At the monthly meeting of the Buffalo Medical Association in June, 1855, I was placed on a committee, as chairman, to report on "the subject of pneumonia, its pathology, prognosis and treatment; together with the diagnostic value of the buffy coat, etc." In accordance with the wishes of the members of the association present at the meeting just mentioned, I had the honor to read in September following, a paper on the "pathology, prognosis and treatment of pneumonia," leaving the "diagnostic value of the buffy coat," as stated at that time, for "a separate report at some future day." My absence from the city during the greater part of the intervening period, must be my excuse for not having in the meantime performed the duty then deferred. Never having intended an indefinite postponement, I beg leave now to present an addendum to my former report, in which I shall endeavor to complete the task assigned to me by the association.

A point preliminary to the consideration of the "diagnostic value of the buffy coat," is to determine the scope of inquiry which this expression was designed to embrace. As it stands in the motion for a committee adopted by the association, June, 1855, it may be understood to refer either to pneumonia alone or to any and all the affections in which the buffy coat is likely to be present. It was probably intended to be taken in the latter sense; at all events I propose to receive it in this broader acceptation. The subject will then resolve itself into the following questions:

1. What are the circumstances involved in the production of the buffy coat?

2. What significance and value belong to it in its pathological relations, and as furnishing therapeutical indications?

The answers to these questions will embrace all the facts and considerations pertaining to the buffy coat which are of interest and importance in a practical point of view.

The circumstances involved in the production of the buffy coat relate to the properties of the element of the blood called fibrin, its quantity, relative and absolute, the phenomena of coagulation, and various extrinsic conditions as well as those pertaining to the other elements of the blood, under which this process takes place.

The fibrinous element of the blood plasma or liquor sanguinis is the only organic constituent which assumes spontaneously a solidified state. It coagulates in consequence of an inherent power, which denotes a vital faculty, inasmuch as the process cannot be accounted for on any known principles of chemistry or physics. The state of coagulation, however, it is to be borne in mind, is abnormal. Fibrin, as it exists in the blood, is a liquid. It is not the same substance after solidification as in its normal condition, and it is not altogether safe to predicate on its characters when coagulated, conclusions respecting those which belong to it when a component part of the circulating fluid. The fibrin in the blood is not coagulated fibrin held in solution. It is fibrin uncoagulated. When once coagulated it does not re-dissolve in the blood plasma from which it has separated itself.

The general appearance which the blood drawn from the vessels by venesection presents after coagulation, is, of course, sufficiently familiar. The coagulating process occupies a period varying from five to twenty minutes. The mass of blood becomes separated into two portions, viz: the coagulum, or clot, and the serum. The former consists of the fibrin, but not of this element alone. It contains all or the greater part of the organized elements of the blood, viz: the colored and colorless corpuscles. These become imprisoned in the meshes of the fibrillating fibrin while the process of coagulation is going on and are retained within the clot, always being more abundant at its lower than at its upper part. The serum holds in solution the various organic elements, exclusive of the fibrin, together with the numerous saline ingredients which enter into the composition of the blood plasma.

The buffy coat, as it is called, is a greater or less portion of the upper part of the clot, consisting of fibrin unmixed with the red corpuscles. It presents the appearance and physical properties which belong to pure coagulated fibrin. It is white, or greyish white, more or less resisting and elastic, and is distinguished by properties determined by microscopical and by chemical examination, which it is not necessary to our present object to consider. This buffy coat may be exhibited by healthy blood under certain circumstances; but, in general, it is to be regarded as an indication of a morbid condition. Now, what are the circumstances involved in its production? These circumstances are intrinsic and extrinsic; in other words, they relate to the blood itself and to exterior influences. We will notice the more important of the circumstances belonging to each class in succession.

The circumstances intrinsically involved in the production of the buffy coat, pertain to the quantity of fibrin which the blood contains, the quantity of red corpuscles and their tendency or otherwise to coherence, the period occupied by the process of coagulation and the density of the serum. These different circumstances may be more or less combined. The most important are the relative proportion of fibrin and red corpuscles and the period occupied by the process of coagulation. An abnormal increase in the quantity of fibrin, diminished number of the red corpuscles, and retarded coagulation, combined, are highly favorable to the production of a buffy coat. This combination obtains in acute inflammations. In pneumonia, for example (and in this inflammation in a marked degree,) the fibrin of the blood is increased, rising from the normal proportion of about 2½ to 7, 8 and even 10 in 1000 parts, according to the researches of Andral and Gavarret, Simon and others. The proportion of red corpuscles is lessened, as is the case uniformly when the quantity of fibrin is increased. The coagulation of the fibrin goes on more slowly than when drawn from a person in health. It is easy to understand how these circumstances contribute to the production of the buffy coat. The retarded process of coagulation gives time for the red corpuscles, which are of greater sp. gr. than the blood-plasma, to subside to the bottom of the clot, leaving more or less of the upper portion entirely free. The greater the abundance of the fibrin and the fewer the number of corpuscles, the more, obviously, will the last-mentioned result take place.

This is the explanation of the production of the buffy coat in inflammations. The fundamental circumstances are those just mentioned. The other circumstances enumerated may, however, coöperate. In proportion to the low specific gravity of the serum, it is evident will be the sinking tendency of the red corpuscles. They will, therefore, gravitate more rapidly when the solid constituents of the blood-plasma are lessened, leaving, consequently, a larger portion of the upper surface free than if the density of the serum were greater. An abnormally diminished sp. gr. of the serum may obtain in inflammation. Again, the red corpuscles appear to exude a viscous fluid, which causes them to adhere together in rows or piles to some extent even in health. This viscous exudation seems to be more abundant in inflammations, so that under these circumstances Robin compares them to grains of flax seed which have been soaked in water. Their coherence is then increased. They run together, presenting, when examined under the microscope, an appearance compared to rouleaux of coin. This aggregation of the corpuscles increases their sinking tendency and thus may contribute to the production of the buffy coat.

If the production of the buffy coat were exclusively incident to the circumstances just stated, it would constitute a diagnostic criterion of acute inflammation. It has been heretofore regarded in this light by practitioners of medicine, and, in fact, only within a few years past have the circumstances involved in its production been understood sufficiently to correct an error, the practical consequences of which have undoubtedly have been highly pernicious.

It is now abundantly ascertained not only that the buffy coat may be formed when acute inflammation does not exist, but even that its production is possible under circumstances intrinsic, or pertaining to the blood, the reverse of those which belong to the inflammatory condition, that is to say, when the proportionate quantity of fibrin, instead of being increased, falls below the normal average. When a notable decrease in the number of red corpuscles takes place, constituting the pathological condition of the blood which characterizes anæmia and chlorosis, assuming the quantity of fibrin to be normal or even somewhat less than normal, more or less of the upper portion of the clot may be devoid of red corpuscles simply because, owing to their paucity, they sink below the surface during the process of coagulation. If, in addition to the diminished number of corpuscles, the plasma be deficient in albumen, and consequently the density of the serum be diminished, as in the instance of Bright's disease, this pathological condition will favor the production of the buffy coat. And under these circumstances its production will be farther favored by any cause, or causes, which retard coagulation. The explanation of its occurrence, irrespective of inflammation, is thus intelligible; and clinical observations have established the fact of its production in cases of different affections in which the relative proportion of fibrin to the red corpuscles is increased not by an absolute augmentation of the former, but as the result of an abnormal diminution of the latter.

The extrinsic circumstances involved in the formation of a buffy coat are to be taken into account. Various circumstances will affect its production irrespective of the condition of the blood, and their mode of operation is chiefly by accelerating or retarding the process of coagulation. The production of the buffy coat will be prevented by extrinsic causes which render the coagulation unduly rapid, even when the intrinsic circumstances are highly favorable; that is, when the quantity of fibrin is increased, or the number of red corpuscles notably diminished. And, conversely, extrinsic causes which prolong the period of coagulation, may occasion a buffy coat in perfectly healthy blood. The correctness of the statement just made has been verified by repeated experiments made by Müller, H. Nasse and Henle, in which an artificial buffy coat was obtained by employing means either for accelerating the sinking of the corpuscles or for retarding the coagulation of the fibrin.*

Of the various circumstances extrinsic to the blood which affect the period of coagulation, the following may be mentioned: The coagulation is less rapid if drawn in a full stream than if the blood flow slowly from a small orifice. In the latter case, a buffy coat will not be likely to be produced under intrinsic circumstances the most favorable. The blood which trickles away from a minute or jagged orifice coagulates almost at once, and the formation of a buffy coat is hardly possible. If the mass of blood after being drawn be agitated, the coagulation takes place sooner than if it had remained at rest. In the latter case a buffy coat might occur, when in the former case it would be prevented.

---

*Lehmann's Physiolog. Chem., vol. i., p. 582.

Observation shows that the blood taken at different periods during the same venesection does not coagulate with uniform rapidity. That which flows last coagulates more rapidly than that which flows first. Hence, if the blood drawn at a single venesection be received into different vessels, the one containing the blood first taken, may present a buffy coat, whilst it is wanting in the one containing the blood which escaped last. It follows from this fact that the quantity of blood drawn at a venesection will affect the production of a buffy coat. If the bleeding be restricted to the abstraction of a few ounces it may be buffed, when it would not have been so had a pint or more been removed.

The size and form of the vessel into which the blood is received affects the period of coagulation. The process goes on more slowly in a deep narrow vessel than in one wide and shallow. The same blood might yield a buffy coat in the former and not in the latter.

Temperature exerts an influence on the process. Blood coagulates sooner in a warm than in a cold vessel. The coagulation of healthy blood may be retarded by cold sufficiently to produce a buffy coat. In the influence of temperature we find the philosophy of the greater efficiency of hot than of cold water applied to a bleeding surface in order to arrest hæmorrhage.

If the vessel which receives the blood presents a rough or irregular inner surface, the coagulation takes place more speedily than if it were regular and smooth. In the dead body the blood in the vessels does not coagulate usually until twelve hours or longer after death. This delay is explained in a great measure by the fact that the lining membrane of the vascular system is so perfectly smooth and polished.

Finally, it is stated by Lehmann that the coagulation of the blood drawn in venesection is retarded by permitting the ligature with which the arm is corded to remain sometime before the vein is opened. This is accounted for by the intrinsic changes which the blood undergoes when it is arrested in its circulation through the veins, and chiefly by the quantity of carbonic acid which, under these circumstances, accumulates. An excess of this constituent of the blood, as is well known, may prevent entirely the formation of a clot. The fluidity of the blood in cases of death by asphyxia is, in part, at least, to be explained in this way.

Having now noticed the more important of the circumstances, intrinsic and extrinsic, involved in the production of the buffy coat, I pass to consider, briefly, the significance and value which belong to it in its pathological relations, and as furnishing therapeutical indications.

I have limited my remarks to the buffed appearance of the clot without reference, as yet, to its consistence and to a character frequently associated, viz: the concavity of the upper surface. When this concavity exists the blood is said to be buffed and cupped. The consistency of the clot and the cupping involve a certain force of contraction inherent in the coagulated fibrin. In proportion to this contractile force, especially in the portion of the clot devoid of red corpuscles, in other words the buffy coat, will the coagulum be found to be firm and resisting, and the depression in the centre of the upper surface or cupped appearance marked. The latter is explained as follows: The presence of the red corpuscles furnishes, of course, a mechanical obstacle to the contraction of the clot. The diameter, therefore, of the space occupied by the buffy coat is less than that of the clot situated below in which the corpuscles are contained. Now the continuity of the superficies of the buffy portion with that of the portion below, limits the contraction here mechanically, while the central portion of the upper surface being less restrained sinks inward. A cupped clot, thus, necessarily involves the existence of a buffy coat; but the converse of this rule is not invariable. The cupped appearance occurs whenever the buffy coat coëxists with sufficient force of contraction in the coagulated fibrin. Formerly much practical importance was attached to this appearance. It was supposed to denote a more intense grade of inflammatory action than the buffy coat alone. I will simply remark that this notion has become obsolete. Cupped as well as merely buffed blood is not peculiar to the morbid condition which obtains in inflammation. It occurs in chlorosis and in connection with other non-inflammatory affections.

What significance and value, pathologically, belong to the buffy coat? The general conclusions to be based on the facts which have been cited, may be summed up in a few words. Under favorable extrinsic circumstances the buffy coat is generally present in acute inflammations, and, other things being equal, is in proportion to the quantity of fibrin above the normal average. Hence it is most constant and marked in those inflammatory affections in which the increased quantity of fibrin is greatest. Of these affections acute articular rheumatism ranks first, and pneumonia second on the list. But it is not peculiar to inflammation. We have seen that its production is not distinctive of "inflammatory blood;" in other words, it does not in all instances denote an abnormal increase in the quantity of the fibrinous element of the blood. Now, are there any characters belonging to the buffy coat incident to inflammation which enable the practitioner to distinguish it from the buffy coat incident to anæmia? to vary the question, is it practicable to discriminate between the buffy coat which denotes an absolute and that which is due to merely a relative excess of fibrin? This inquiry I have reserved for the present connection. Its bearing on the "diagnostic value of the buffy coat," is sufficiently obvious. The discrimination may generally be made by reference to the size of the clot, in connection with its consistence. *A large firm clot which is buffed and cupped is highly significant of inflammation, or, in other words, of an absolute increase of fibrin. On the other hand, the buffed and cupped clot incident to blood which is impoverished,* i. e., *deficient in red corpuscles, without any increase, and it may be even with decrease of fibrin, is small in size, and perhaps, as a rule, less consistent.**

But we have seen that the production of the buffy coat is affected by numerous extrinsic circumstances which are not to be overlooked in endeavoring to set forth, in general terms, its pathological significance and value. Assuming, on the other hand, the fibrin to be increased, and, on the other hand, the red corpuscles to be notably diminished, the production of the buffy coat will depend on the quantity of blood removed, the size of the stream, the capacity and form of the receiving vessel, the smoothness of the inner surface of the latter, the temperature, a state of repose or agitation, etc. —circumstances which affect the period occupied by the process of coagulation. If more or less of these extrinsic circumstances are unfavorable, the buffy coat may be wanting, although the condition of the blood itself be favorable for its production. It is obvious that the appearances of the clot cannot be considered as representing the pathological condition of the blood, without a due estimation of these various contingencies. Blood which is truly inflammatory may not present the buffy coat in consequence of the non-concurrence of extrinsic circumstances. A series of observations in which the depth of the buffy portion of the clot, the amount of cupping, etc., are noted, it is clear are of small practical value unless the extrinsic circumstances influencing the process of coagulation are uniform, and it must be at once evident that it is difficult, if not indeed impracticable, to secure uniformity in this regard.

These considerations certainly divest an examination of the blood with reference to the buffy coat of much of the clinical importance heretofore attributed to it. They do not by any means render nugatory the positive in-

---

* The different appearances which the clot presents as regards size and form in health, in inflammation and in anæmia, are figured in Williams Principles of Medicine, page 135.

ferences to be drawn from the existence of the buffy coat when present, but they interfere with deductions from the fact of its absence, and with regarding the buffy coat as a measure, quantatively, of the pathological condition of the blood which it represents. Moreover, inasmuch as a very small quantity of blood cannot serve as a fair test of the tendency to the production of the buffy coat, as a diagnostic criterion it is open to the objection that it is only available after an important therapeutical measure, (bloodletting) has been resorted to.

In conclusion, what therapeutical indications are furnished by the production of a buffy coat? I will narrow this inquiry by assuming it to be ascertained that the buffy coat is due to an absolute increase of fibrin, that is to say, to an acute inflammation. Thus narrowed, were I to pursue the topics which cluster around the inquiry, they would lead to a wide range of discussion. I shall devote to it but a few words, the more because it is an inquiry rather relevant to the subject of this report than distinctly embraced within it. I assume the existence of a buffy coat and that it be ascertained to denote a corresponding augmentation of the fibrin of the circulating blood. I will go a step farther and instance a disease in which the fibrin has been found to be augmented in a marked degree, in acute pneumonia, or, if the term be preferred, sthenic pneumonia. What therapeutical indications in this disease are based on the knowledge of the fact that fibrin of the blood is increased from 2½ to 7, 8, 9 or 10 parts in 1000? Does the abnormal increase of fibrin stand in a causative relation to the local inflammation? Ascertained facts do not warrant the affirmative to this question. It is not determined that the increase of fibrin precedes the development of the inflammation. Clinical observations only teach us that the two are concomitant. In what, the nature of the pathological connection existing between the local phenomena and the condition of the blood is not known. We cannot, therefore, theoretically say of how much importance it would be to effect a reduction in the excess of the fibrin of the blood. Assuming, however, that this is a desirable end, then, the questions arise, can it be accomplished, and, if so, by what means? Were we to consider these questions fully we should probably find that our ability to effect by therapeutical agencies a reduction in the fibrinous constituent of the blood during the continuance of the inflammatory process, is, to say the least, as uncertain as the desirableness of the object. I shall notice, however, but one therapeutical agent as regards its effect in this direction, viz: bloodletting. The influence of blood-letting on the quantity of fibrin contained in the blood is readily ascertained and may be settled by abundant observations. *It increases the amount of fibrin.* M. Bernard says: "Bleed an animal very often, each time removing the fibrin from what you have withdrawn, and injecting again the rest, the nourishment continuing the same as before the experiment, the strange fact will be observed that the quantity of fibrin goes on increasing. M. Majendie, who made the trial, said that this was the cause of finding more fibrin in cases of inflammation because they were treated by bloodlettings."

The number of red corpuscles is notably diminished by bloodletting. Hence, it is obvious that a direct result of this therapeutical agent is a tendency to the development or the increase of the buffy coat. It is needless to remark that if it be a desirable end in the treatment of pneumonia, as well as any other local inflammation, to seek to reduce the quantity of fibrin in the blood, bloodletting, so far from promoting this end, tends to counteract it. The value of bloodletting in inflammation of the lungs or other organs, must rest on other ground. And it is obvious that a practitioner who regards the presence of the buffy coat as an indication for the lancet (as has been the case in times past) will be likely to find greater occasion for the use of this instrument on each successive venesection.

May 24, 1856.

The report was accepted.

After some discussion as to the proper disposal of the various reports of the different members of the committee, it was moved by Dr. White,

"That the several reports of this committee be accepted and the committee discharged, and that the thanks of the association be tendered to the several gentlemen for their able and interesting reports." Carried.

*Prof. White* then moved, with the request that the motion should lie on the table for one month, "That the association adopt the report of the chairman of the committee."

Ordered to lie on the table for one month.

*Prof. Hadley* inquired, in reference to the statement in Dr. Flint's report, that coagulated fibrin, as found in blood outside the body, was not to be considered as identical with liquid fibrin as it existed within the circulation. Did we know anything positive as to the real existence of fibrin *as* fibrin in the blood? Is not coagulation a process of decomposition, and can we judge of the properties of fibrin within the circulation, from the changes it undergoes when withdrawn from the body?

Dr. Flint replied that he had said that coagulated fibrin was to be distinguished from liquid fibrin, that the former is an abnormal, the latter a normal form of this element, and that we are not able to learn the properties of liquid fibrin from the phenomena of coagulation.

*Dr. Hadley* exhibited a specimen of the new metal aluminum, and stated that it is now made in this country, that a chemist of New Jersey has discovered a plan of manufacturing sodium by a continuous process like that for procuring zinc, and that the sodium thus made can be furnished for less than a dollar a pound; thus cheapening very much the manufacture of aluminum. The specimen exhibited was a light metal, relatively about one quarter as heavy as silver, having about the lustre of block tin. It was malleable, moderately ductile, tough, and about as hard as silver.

On the inquiry of the President as to prevailing diseases, no epidemic was reported; the diarrhœa reported last month had mostly subsided, and the city was stated to be healthy.

*Dr. Hunt* reported a case of strangulation, in which, with Drs. Wilcox and Baker, he had been called to make a post-mortem examination. A large piece of beef-steak was found covering the opening of the glottis. The man had been subject to choking when at meals during all his life, which was not surprising if he often swallowed pieces of meat as large as that found in his pharynx. It was remarkable, however, that his father had been equally subject to fits of choking, and that an uncle had died in the same manner.

*Dr. Flint* moved that a committee of three be appointed to select for the association certain German, French and English periodicals, and that a fund should be raised for that purpose.

He subsequently modified his motion to instruct this committee to coöperate with one from the County Medical Society.

The motion was then carried, and the President appointed Drs. Flint, Wyckoff and Wilcox, as such committee.

*Dr. Newman* suggested that the Registrar-General's Reports, published by the British Government, were extremely valuable, and wished that some steps might be taken to procure them.

And the association then adjourned.

SANFORD B. HUNT, Secretary.

